# Granulocyte colony-stimulating factor-producing cutaneous squamous cell carcinoma: A case report and literature review

**DOI:** 10.1097/MD.0000000000042798

**Published:** 2025-06-06

**Authors:** Akihiro Ishiguro, Nobuhiko Iwashita, Megumi Otani, Daisuke Watanabe

**Affiliations:** a Department of Dermatology, Aichi Medical University, Nagakute, Aichi, Japan.

**Keywords:** G-CSF-producing tumor, poor prognosis, squamous cell carcinoma, tumor marker, verrucous carcinoma

## Abstract

**Rationale:**

Granulocyte colony-stimulating factor (G-CSF) leads to an abnormal increase in the white blood cell count and is associated with a poor prognosis due to accelerated progression of the G-CSF-producing tumors. G-CSF-producing tumors arising from skin malignancies are rare.

**Patient concerns:**

A 72-year-old female patient presented with a 6-cm skin tumor on the genital midline. Two biopsy specimens revealed condyloma acuminatum, but a total resection and bilateral inguinal lymph node dissection were performed because the tumor was suspected of being malignant.

**Diagnoses:**

Histopathological analysis revealed poorly differentiated verrucous carcinoma with a lymph node metastasis. After the white blood cell count and G-CSF rose to 111,000/μL and 543 pg/mL, respectively, the tumor was diagnosed as a G-CSF-producing tumor. The patient died 8 months after her initial visit.

**Interventions:**

We summarize the previously reported cases of G-CSF-producing cutaneous squamous cell carcinoma (SCC).

**Outcomes:**

The present report includes a review of 11 previous cases of G-CSF-producing cutaneous SCC, all of which occurred in Japanese patients. Compared with the typical SCC patient, the patients in the studies cited were relatively young, and the lesions tended to occur on the genitals or buttocks. The mean tumor length is 9.2 ± 4.8 cm, and many instances were much larger than typical cutaneous SCCs due to the very rapid growth of G-CSF-producing tumors. The histological type of these cases included poorly differentiated SCC and a special type of SCC with high malignancy potential. Only 2 of the 11 patients survived; the median overall survival of the deceased patients was 7.8 ± 6.4 months, which was indicative of an extremely poor prognosis.

**Lessons:**

The present case is the first to report a case of G-CSF-producing tumors developed from verrucous carcinoma and had an extremely poor prognosis. G-CSF-producing cutaneous SCC should be considered in the differential diagnoses of malignant lesions developing on the genitals or buttocks or having a histological type or growth rate indicating high malignancy.

## 1. Introduction

Granulocyte colony-stimulating factor (G-CSF)-producing tumors (GPT) are frequently associated with lung, esophageal, stomach, thyroid, and bladder malignancies, but there are few reports of their association with skin malignancies.^[1]^ G-CSF leads to an abnormal increase in the white blood cell count (WBC) and is associated with a poor prognosis due to accelerated progression of the GPT. The diagnostic criteria for GPT are: (i) severe leukocytosis mainly due to mature granulocytes in the peripheral blood; (ii) elevation of serum G-CSF levels; (iii) normalization of leukocytosis and serum G-CSF after tumor resection or treatment; and (iv) histological evidence of a G-CSF-producing tumor by immunohistochemistry. Verrucous carcinoma (VC) is usually a low-grade malignancy, but a previous study found that its development into GPT worsens the prognosis.^[2]^ The patient provided informed consent for the publication of this case.

## 2. Case report

**Figure 1.**
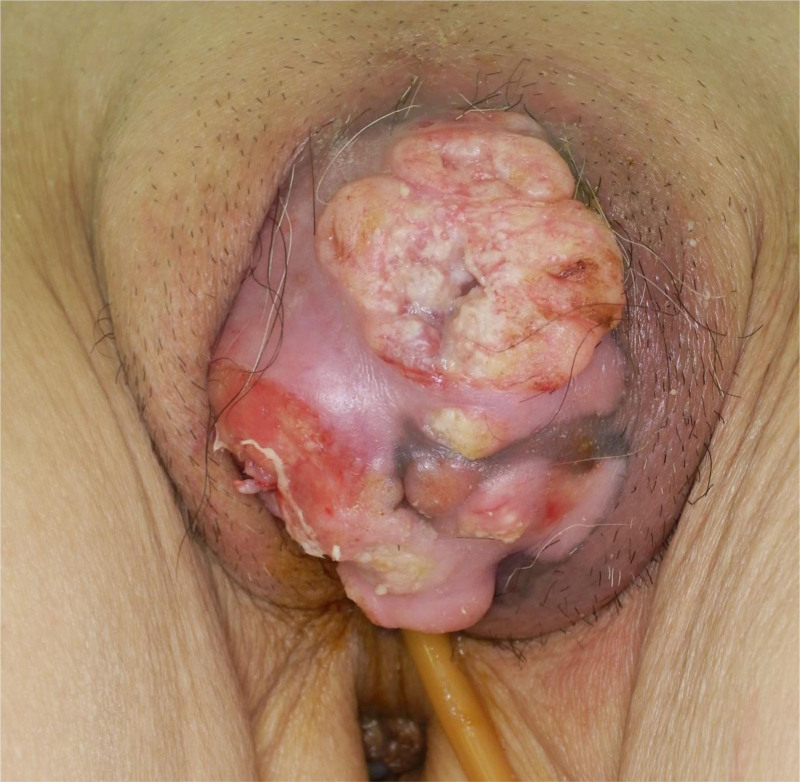
Clinical findings at the initial visit. A 6-cm skin tumor with poor mobility due to subcutaneous infiltration was observed in the genital midline. The surface consisted of smooth and cauliflower-like raised areas with bleeding.

**Figure 2.**
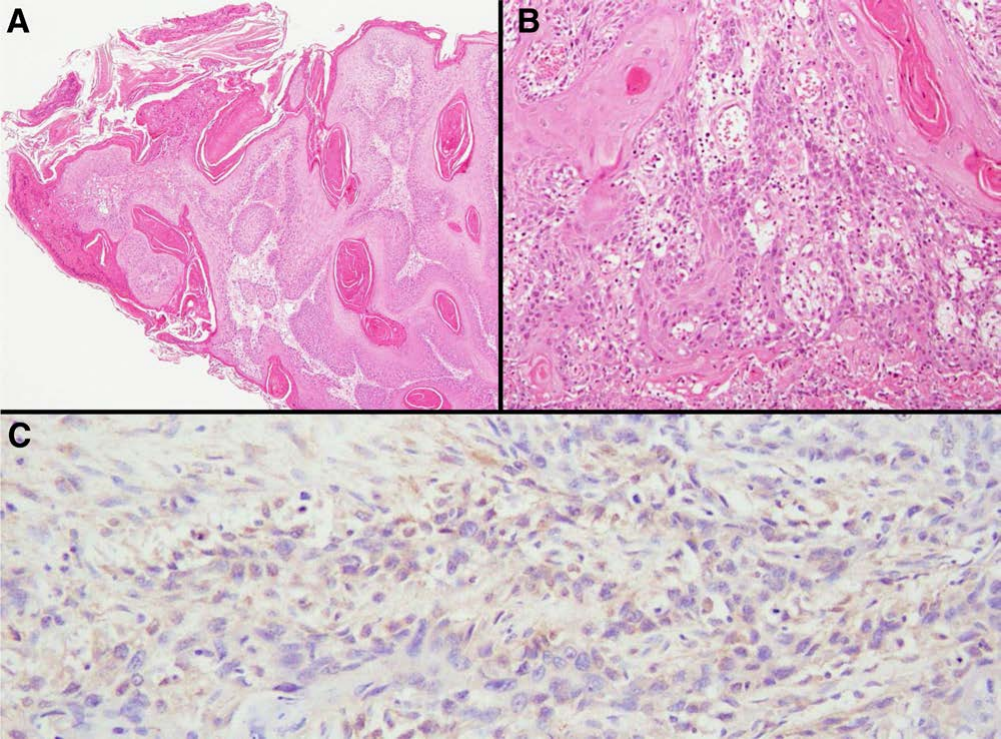
Pathological findings of a total resection specimen. (A) The tumor surface demonstrated proliferation of non-dysplastic squamous cells, a finding consistent with condyloma acuminatum (hematoxylin and eosin, magnification × 10). (B) Atypical squamous cells can be seen infiltrating in a cord-like manner with keratinization, a finding consistent with poorly differentiated SCC (×200). (C) Immunohistochemical staining with anti-G-CSF monoclonal antibody showed a weak positive reaction in the cytoplasm of atypical tumor cells (×200). G-CSF = granulocyte colony-stimulating factor, SCC = squamous cell carcinoma.

**Figure 3.**
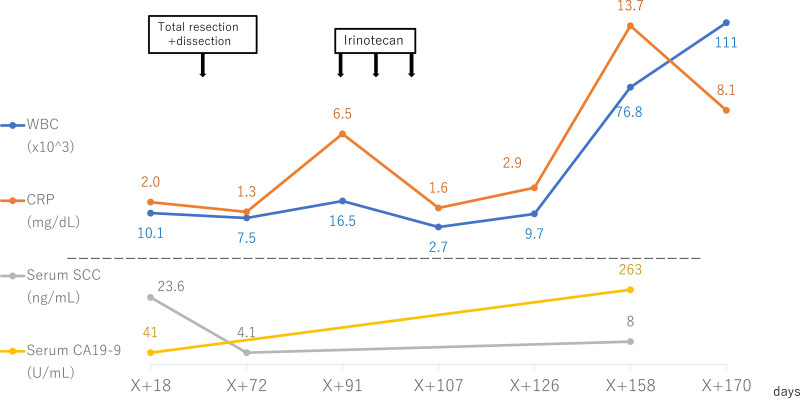
The serum white blood cell (WBC) count, CRP, serum SCC, and serum CA19-9 during the clinical course. X indicates the initial visit. X + 45 indicates the timing of the total resection and bilateral inguinal lymph node dissection. X + 91, 98, 105 indicate the timing of irinotecan 100 mg/m^2^ administration. CRP = C-reactive protein, SCC = squamous cell carcinoma.

A 72-year-old, female patient with a history of bladder cancer presented to a dermatology clinic with a swelling and papillary growths of the clitoris 50 days before her initial visit. Analysis of a biopsy specimen in a dermatology clinic revealed condyloma acuminatum. Based on the clinical findings and the presence of bilateral inguinal lymph node swellings, malignant carcinoma was suspected, and she was referred to the study center. The clinical findings on presentation demonstrated a 6-cm skin tumor on the genital midline with poor mobility due to subcutaneous infiltration (Fig. 1A). The surface consisted both of smooth areas and cauliflower-like, raised areas. A second biopsy confirmed the initial finding of condyloma acuminatum. Computed tomography (CT) demonstrated bilateral inguinal lymph node swellings and osteolytic changes in the left pubic bone. The genital tumor was considered a malignant carcinoma and was resected with a 1-cm margin. A bilateral inguinal lymph node dissection was then performed. Histopathological analysis revealed squamous cell carcinoma (SCC) and bilateral inguinal lymph node metastases. Pathological analysis found SCC with well-differentiated and poorly differentiated areas (Fig. 2A, B). Human papillomavirus (HPV) immunostaining and HPV-PCR returned negative. G-CSF immunostaining, which was performed later, was positive (Fig. 2C). The value for serum SCC, a tumor marker, was 23.6 preoperatively and decreased to 4.1 postoperatively. Figure 3 shows the progress of WBC and CRP, serum SCC, serum CA19-9 during the clinical course. Seventy-eight days after the initial visit, positron emission tomography (PET)-CT revealed multiple lung metastases and a metastasis to the left pubic bone. Ninety-one days after the initial visit, 1 course of irinotecan 100 mg/m^2^ was administered once weekly for 4 weeks as systemic therapy, then was followed by a 2-week rest period. The therapy was discontinued due to adverse events, including diarrhea and fatigue. On day 156 after the initial visit, the patient was admitted for empyema. The WBC count remained high at 76,800/μL, and CRP was 13.7 mg/dL even after improvement of the empyema. G-CSF was 543 pg/mL (reference value < 39). Based on these findings, GPT was diagnosed. The value for tumor marker CA19-9 was 41 U/mL (reference value < 37 U/mL) preoperatively but rose to 263 U/mL after empyema development. On day 178 after the initial visit, the patient died.

## 3. Discussion

### 3.1. Differentiating between the Buschke–Löwenstein tumor and VC

VC is a giant, malignant tumor with an exophytic, cauliflower-like, whitish-yellow appearance.^[3]^ Buschke–Löwenstein tumor (BLT) is a sexually transmitted disease stemming from an HPV infection and begins as a benign tumor.^[4]^ VC and BLT are sometimes considered the same disease, but some experts argue that they are distinct.^[3,4]^ They may have similar clinical and pathological findings but a completely different prognosis, lending strength to the idea that they are distinct, nosological entities^[5,6]^ (Table 1). Pathological examination for HPV and koilocytosis is reportedly useful in differentiating the 2 conditions.^[3]^ The correct diagnosis in the present case was VC rather than BLT. In general, the former is unlikely to metastasize and progresses slowly. Our patient, who had a rare case of poorly differentiated VC, died 8 months after her initial symptoms. Even if the diagnosis is difficult to make on the basis of clinical and pathological findings, a total resection is necessary if a malignancy is suspected.

**Table 1:** Differences between the Buschke–Löwenstein tumor and verrucous carcinoma (there are differing views on the definition of the diseases).

	Buschke–Lowenstein tumor^[4]^	Verrucous carcinoma^[3]^
Common age	Forties	Elderly
Clinical finding	Slow growth, verrucous tumor	Verrucous tumor
Malignancy	About half of cases become malignant^[5,6]^	Well-differentiated SCC, almost no metastasis
HPV type	6, 11	None (rarely 16)
Pathology	Koilocytosis, hyperplastic basal cell abscesses in the stroma	Large spinous cells containing dyskeratotic cells, intraepithelial micro-abscesses

SCC = squamous cell carcinoma.

### 3.2. Review of previous cases of G-CSF producing cutaneous squamous cell carcinoma

SCC is a common, histological type of GPT.^[1,7]^ GPT has also been reported in malignant melanoma and angiosarcoma in a burn scar on his buttock, but cutaneous SCC (CSCC) is the most common in skin malignancies.^[8,9]^ Table 2^[7,10–18]^ summarizes the age, sex, primary site, latency period (months from onset to diagnosis based on appearance), tumor size (maximum diameter in cm), WBC count (×10^3^/μL), G-CSF (maximum value during the clinical course in ng/L), serum SCC antigen (maximum value during the clinical course in ng/mL), and overall survival (defined as the duration from SCC diagnosis to death in months) of 11, previous cases of G-CSF-producing cutaneous squamous cell carcinoma (GCS), including the present case, along with the associated serum G-CSF value.

**Table 2:** Clinical characteristics of past cases of G-CSF-producing cutaneous squamous cell.

Case	Year reported	Author	Age	Sex	Site	Histological type	Latency period (months)	Size (cm)	WBC (×10^3^/μL)	G-CSF (ng/L)	Serum SCC (ng/mL)	OS (months)
1	1999	Kato^[10]^	58	M	Heel	SCC (Scar)	14	5.5	57.1	39.2	Normal	13
2	2003	Fujio^[11]^	74	M	Forehead	Pseudo-angiosarcomatous SCC	3	2	66.4	81.7	NA	20
3	2007	Nara^[12]^	63	M	Brachium	Undifferentiated SCC	120	10	20.6	117	40	5
4	2011	Ito^[13]^	90	F	Vulva	Well-differentiated SCC	120	6	36.7	430	5.2	14
5	2013	Yamasaki^[14]^	81	M	Cheek	SCC with epithelial–mesenchymal transition	36	9	26.3	307	NA	Alive
6	2014	Nakamura^[15]^	70	F	Buttocks	SCC	12	6.2	74.1	357	Normal	6
7	2015	Mizugaki^[16]^	69	M	Back	SCC with a rhabdoid phenotype	60	15	32.4	118	Normal	5
8	2018	Harafuji^[7]^	76	F	Vulva	Well-differentiated SCC	24	19	125	394	30	2
9	2020	Eto^[17]^	67	M	Dorsum of hand	Well-differentiated SCC	60	10	54.8	403	NA	Alive
10	2022	Mizuno^[18]^	53	M	Buttocks	SCC (decubitus)	48	12	53.1	248	NA	1
11	2024	Ishiguro	72	F	Vulva	Poorly differentiated SCC	3	6	111	543	23.6	4
Mean ± SD			70 ± 10				45.4 ± 42.2	9.2 ± 4.8	69.8 ± 33.4	276.2 ± 166.6		7.8 ± 6.4*

G-CSF = granulocyte colony-stimulating factor, NA = not available, OS = overall survival, SCC = squamous cell carcinoma, SD = standard deviation, WBC = white blood cell.

*Nine patients excluding the survivors.

All the 11 cases reviewed were reported in Japan. Genetic or HLA-related differences may account for the apparent preponderance of Japanese patients, but the precise reason is still unknown. More than 80% of typical CSCC cases, but less than half of GCS cases, occur in patients older than 70 years. Patients with GCS tend to be relatively young, but their age varies widely. CSCC frequently develops in the head or neck area, but 5 of the 11, previously reported cases occurred on the genitals or buttocks, suggesting that chronic irritation, rather than ultraviolet radiation exposure, a common etiology of CSCC, may be the cause.

Six of the eleven cases of CSCC reviewed were SCC with either unknown or well-differentiation and included a large proportion of malignant lesions comprising both the special or poorly differentiated types. A previous study reported that 26 of 65 GPT cases (40%) reported in Japan were of SCC.^[7]^ Many studies reported that the risk of GPT development rose as the level of differentiation decreased. Horii et al reported that recurrences, which may be less differentiated than the original lesion, may transform into GPT.^[19]^ In the present review, the development of SCC from scars^[10]^ and decubitus ulcers,^[18]^ which are known to be highly malignant, and the presence of highly malignant types, such as the rhabdoid type^[16]^ and pseudo-angiosarcomatous SCC,^[11]^ may be related to this transformation into a GPT. Koebnerized SCC is a quickly growing keratotic nodule that shares clinical characteristics with GCS. Koebnerized SCC has been suggested that surgery or other traumatic stimuli may influence the physiological processes of cancer development.^[20]^

The latency period from onset to SCC diagnosis is 45.4 ± 42.2 months on average (mean ± standard deviation) but varies widely among individual cases possibly because the GPT was present from the outset in some patients while the tumor transformed into a G-CSF-producing lesion during the disease course in other patients. In the present instance, the tumor had a short latency period and grew rapidly from onset, suggesting that it may have been a GCS lesion from the start. Cases with a long latency period in which the tumor progresses rapidly following a recurrence or metastasis may transform into a GPT during the disease course. The mean tumor length is 9.2 ± 4.8 cm, and many instances were much larger than typical CSCC due to the very rapid growth of GPTs and the inclusion of cases that had been left untreated for a long time.

The WBC count was significantly higher the reference value in all the reviewed cases. Serum G-CSF may be measured if a cause other than an infection is suspected. The G-CSF value varies depending on when it is measured. The present case had the highest serum G-CSF value among the cases reviewed. The period from onset to death was relatively brief at 7 months, and it is possible that the extreme malignancy of the lesion was related to the high level of G-CSF. In the all the reviewed cases, a positive correlation was found between the WBC count and serum G-CSF (Pearson correlation coefficient: 0.49) (SPSS Statistics 29.0 for IBM, NY). Furthermore, the G-CSF value in case 1 was slightly above the upper limit (32.7 ng/L) of the reference value.^[10]^ However, there are various, possible causes of elevated G-CSF and leukemoid reaction besides a GPT, such as a bacterial infection, myeloproliferative disease, and bone marrow metastasis.^[19,21]^ Immunohistochemical analysis of the G-CSF was not performed in case 1, and multiple metastases had already developed before the increase in serum G-CSF was detected; thus, it is possible that the tumor was not producing G-CSF. Improvement in the response to treatment is one of the diagnostic criteria for GPT.^[2]^ However, patients with GPT are often unable to receive the requisite treatment due to a metastasis, recurrence or rapid progression. Therefore, an abnormally high WBC count, G-CSF value and the fact that the tumor itself is producing the G-CSF should be proved.

Serum SCC, a tumor marker, was measured in 7 cases, and was found to be elevated in 4 cases (57.1%). However, serum SCC was not necessarily measured at the same time as G-CSF, and the SCC level before the resection of the primary tumor may have been included. The measurement of tumor markers is not consistently useful in SCC and is therefore not routinely recommended. Nara et al reported that SCC and G-CSF values changed in parallel.^[12]^ However, in the present case, the SCC value was already high before resection, and when the serum G-CSF increased, the CA19-9 value increased much more noticeably than the SCC value possibly due to a change in the characteristics of the tumor after its transformation into a G-CSF-producing lesion. It has been reported that tumor phenotypes may ***swith during disease progression or in chemotherapy.^[22,23]^

GCS is a malignant carcinoma with a very poor prognosis. Only 2 of 11 patients (18%) survive. In the present review, the surviving patient (case 9) received the diagnosis of GCS before metastasis^[17]^ and the other surviving patient (case 5) is a rare case in which metastasis disappeared and G-CSF values decreased with TS-1 therapy (a preparation combining tegafur, gimeracil, and oteracil potassium).^[14]^ The mean duration from diagnosis to death in the other, 9 cases was very brief, at 7.8 ± 6.4 months. The patients normally died within a few months after an increase in the WBC count raised the suspicion of GCS. G-CSF is reportedly an important prognostic factor of malignant tumors^[24]^; the GPT itself expresses G-CSF receptors, which promote the proliferation, invasion, and metastasis of tumor cells.^[25]^ Furthermore, G-CSF was found to inhibit the antitumor immune response by inducing immunosuppressive cells.^[26]^ Inhibition of the G-CSF receptor has been shown to mitigate immunosuppression in viral infections, suggesting that its therapeutic potential in the microenvironment of GPT warrants further investigation.^[27]^ Most cases in this review, including the present one, responded poorly to systemic therapy. Thus, early detection based on blood analysis findings and evidence of abnormal accumulation in the bone marrow on PET-CT are important for the successful treatment of a GPT.^[17]^

## 4. Conclusion

VC generally progresses slowly, unlike the present case. The present case is the first to report a case of GCS developed from VC with an extremely poor prognosis. GCS should be considered in the differential diagnoses of malignant lesions developing on the genitals or buttocks or having a histological type or growth rate indicating high malignancy. Early detection of a GCS using WBC, PET-CT, and tumor markers may improve the prognosis.

## Acknowledgments

We thank Dr Akira Shimizu and Dr Reimon Yamaguchi (Department of Dermatology, Kanazawa Medical University) for HPV immunostaining and PCR.

## Author contributions

**Conceptualization:** Akihiro Ishiguro.

**Data curation:** Akihiro Ishiguro.

**Formal analysis:** Akihiro Ishiguro.

**Investigation:** Akihiro Ishiguro.

**Methodology:** Akihiro Ishiguro.

**Project administration:** Akihiro Ishiguro.

**Supervision:** Nobuhiko Iwashita, Daisuke Watanabe.

**Validation:** Akihiro Ishiguro.

**Visualization:** Akihiro Ishiguro.

**Writing – original draft:** Akihiro Ishiguro.

**Writing – review & editing:** Akihiro Ishiguro, Nobuhiko Iwashita, Megumi Otani, Daisuke Watanabe.
